# Causal Association Between Tea Consumption and Kidney Function: A Mendelian Randomization Study

**DOI:** 10.3389/fnut.2022.801591

**Published:** 2022-03-29

**Authors:** Yangchang Zhang, Yang Xiong, Shisi Shen, Jialu Yang, Wei Wang, Tingting Wu, Li Chen, Qiuhua Yu, Hangjia Zuo, Xu Wang, Xun Lei

**Affiliations:** ^1^School of Public Health and Management, Chongqing Medical University, Chongqing, China; ^2^Research Center for Medicine and Social Development, Chongqing Medical University, Chongqing, China; ^3^The Innovation Center for Social Risk Governance in Health, Chongqing Medical University, Chongqing, China; ^4^Research Center for Public Health Security, Chongqing Medical University, Chongqing, China; ^5^The West China Hospital, Sichuan University, Chengdu, China; ^6^The First School of Clinical Medicine, Chongqing Medical University, Chongqing, China; ^7^Chongqing Collaborative Innovation Center for Functional Food, Chongqing University of Education, Chongqing, China; ^8^School of Public Health & Institute of Child and Adolescent Health, Peking University, Beijing, China; ^9^Department of Critical Care Medicine, Peking Union Medical College Hospital, Peking Union Medical College and Chinese Academy of Medical Sciences, Beijing, China

**Keywords:** tea consumption, chronic kidney disease (CKD), SNPs, albuminuria, Mendelian randomization

## Abstract

**Background:**

Causal research concerning the consumption of tea and the risk of chronic kidney disease (CKD) is limited. This study identified the potential causal effects of tea intake on CKD, the estimated glomerular filtration rate (eGFR), and albuminuria.

**Methods:**

Genome-wide association studies (GWASs) from UK Biobank were able to identify single-nucleotide polymorphisms (SNPs) associated with an extra cup of tea each day. The summary statistics for the kidney function from the CKDGen consortium include 11,765 participants (12,385 cases of CKD) and 54,116 participants for the urinary albumin-to-creatinine ratio who were mostly of European descent. A two-sample Mendelian randomization (MR) analysis was performed to test the relationship between the selected SNPs and the risk of CKD.

**Results:**

A total of 2,672 SNPs associated with tea consumption (*p* < 5 × 10^–8^) were found, 45 of which were independent and usable in CKDGen. Drinking more cups of tea per day indicates a protective effect for CKD G3-G5 [odds ratio (OR) = 0.803; *p* = 0.004] and increases eGFR (β = 0.019 log ml/min/1.73 m^2^ per cup per day; *p* = 2.21 × 10^–5^). Excluding two SNPs responsible for directional heterogeneity (Cochran *Q p* = 0.02), a high consumption of tea was also negatively correlated with a lower risk of albuminuria (OR = 0.758; *p* = 0.002).

**Conclusion:**

From the perspective of genes, causal relationships exist between daily extra cup of tea and the reduced risk of CKD and albuminuria and increased eGFR.

## Introduction

Chronic kidney disease (CKD) is an increasingly serious public health issue, which affects 9–16% of the world’s population (1). Globally, the number of deaths attributed to CKD has increased to approximately 1.2 million, and the mortality rate in all ages has increased by 41.5% from 1990 to 2017 (2). The time-series model from the global burden of disease (GBD) group indicated an increasing trend in the number of years of life lost, from around 26 million annually in 2016 to 52.5 million in 2040 (3). Although treatment has been shown to slow the progression, CDK may proceed at different paces over time (4). CKD can progress to kidney failure and early cardiovascular disease in the end stage, namely, end-stage renal disease (ESRD). Dialysis or kidney transplant is necessary for survival on account of dysfunctional kidneys (4). CKD is associated with an increased risk of many other conditions, including cognitive impairment, renal bone disease, chronic anemia, and death by sepsis and cardiovascular disease (5, 6). Empirical studies have found that lifestyle factors, such as smoking, alcohol consumption, and obesity, are related to a higher risk of the disease (7–9). Aside from these factors, an increasing interest is being paid to the important role of diet (10). As an essential part of the diet, beverages can influence the maintenance of general health and renal function and inhibit/abet the high-risk factors that may lead to CKD (e.g., hypertension, obesity, and diabetes) (10, 11).

Tea is one of the most popular beverages in the world (12). Many longitudinal and cross-sectional studies have investigated the association between tea consumption and the risk of CKD and the estimated glomerular filtration rate (eGFR), and studies on the association between tea intake and albuminuria are limited (12–16). Nevertheless, these studies failed to reach a robust and consistent conclusion. This discrepancy may be due to diverse tea types (e.g., green, black, and oolong tea) and demographic heterogeneity (e.g., sex) (16). For example, no evidence showed that extra tea intake could improve renal function or delay the stage of nephropathy in Iran, Singapore, and Netherlands (12, 14, 16). However, another follow-up survey reported that tea intake can exert positive effects on patients with metabolic syndrome (MetS) (13). Additionally, a study from China found that oolong tea could obviously promote the efficiency of eGFR compared to black tea and green tea (15). In view of the inconsistent results and different indicators of previous studies, the effects of tea consumption on the different indicators of renal function need to be investigated. Additionally, considering the limitations of observational studies and the potential influence of confounding factors or reverse causality, a causal relationship between tea consumption and the risk of abnormal CKD indicators cannot be concluded. Therefore, whether a causal relationship exists between tea intake and kidney function remains unclear.

Mendelian randomization (MR) is a genetic epidemiology design, which improves the power of causal inference by applying proxy germline genetic variants as instrumental variables for exposure (e.g., tea intake) on an outcome (e.g., CKD) (17). Single-nucleotide polymorphism (SNP) sites are randomly assigned in the beginning, so the bias of reverse causation and residual confounding is avoided (18). The two-sample MR design has not yet been used to determine the causal association between tea intake and the risk of abnormal renal function. Thus, a conventional MR method was performed in this study to estimate whether tea intake is causally associated with the risk of abnormal renal function (e.g., CKD, eGFR, and albuminuria).

## Materials and Methods

### Genetic Instrument Selection

The genome-wide association study (GWAS) summary data set for tea consumption (Phenotype Code:1488_raw) based on UK Biobank was downloaded from Neale Lab^1^ (GWAS round 2), including over 349,376 samples of European ancestry. GWAS was adjusted for sex, age, age^2^, sex × age, sex × age^2^, and first 20 ancestry principal components. The data of habitual tea consumption were obtained as a baseline from a dietary questionnaire. In the questionnaire, the following question was asked: “How many cups of tea do you drink each day (including black and green tea)?” GWAS summary statistics for unconverted daily tea consumption were used to identify SNPs associated with tea drinking. Detailed information regarding the phenotype and the process of quality control in UK Biobank is available on the Neale Lab website.^2^ We selected autosomal biallelic SNPs with *p* < 5 × 10^–8^ and performed further quality control based on minor frequency >1%, ending up with 2,672 unique SNPs. Furthermore, based on the European sample reference data from the 1000 Genomes Project (19), we clumped these 2,672 SNPs with linkage disequilibrium *r*^2^ < 0.001 at a 10,000 kb window, confirming the independence of the selected genetic variants. Finally, 45 independent SNPs were associated with tea consumption. The proportion of variance in tea consumption explained by each SNP was estimated using the *R*^2^ value (20), and the instrumental strength of each SNP was assessed using the *F*-statistic (21). Detailed information on the relationship between the selected SNPs and exposures is shown in Table 1.

**TABLE 1:** Characteristics of SNPs associated with tea consumption.

SNP	Position	EAF	EA	BETA	SE	*P*	N	*R* ^2^	*F*-statistic
rs1030510	7:17100273	0.45	G	–0.0436	0.0069	3.6E-10	349376	0.000114	40
rs10741694	11:16286183	0.63	C	0.0404	0.0071	1.53E-08	349376	0.0000927	32
rs11022751	11:13307613	0.27	C	0.0497	0.0078	1.83E-10	349376	0.000116	41
rs112476491	7:17204040	0.03	A	–0.1186	0.0194	8.88E-10	349376	0.000107	37
rs11487328	1:174601659	0.38	C	–0.0493	0.0071	5.16E-12	349376	0.000138	48
rs11636222	15:75515312	0.23	G	–0.0557	0.0089	3.79E-10	349376	0.000112	39
rs12591786	15:60902512	0.16	T	–0.0609	0.0096	2.32E-10	349376	0.000115	40
rs12600469	17:40834073	0.62	T	0.0406	0.0071	1.22E-08	349376	0.0000936	33
rs12901092	15:75374145	0.39	A	–0.0654	0.0071	3.2E-20	349376	0.000243	85
rs12916473	15:75321999	0.04	A	0.1233	0.0185	2.63E-11	349376	0.000127	44
rs140775622	20:62962869	0.17	T	0.0707	0.0099	9.33E-13	349376	0.000146	51
rs1481012	4:89039082	0.11	G	–0.0778	0.0109	9.41E-13	349376	0.000146	51
rs149375687	5:152034989	0.27	T	–0.0449	0.0078	7.26E-09	349376	0.0000948	33
rs1601409	12:17066769	0.46	G	0.0382	0.0069	3.67E-08	349376	0.0000877	31
rs1669433	12:11349732	0.84	G	0.0551	0.0093	3.33E-09	349376	0.0001	35
rs17645813	7:17419697	0.08	A	–0.1058	0.013	3.32E-16	349376	0.00019	66
rs199621380	1:150700614	0.41	G	0.0413	0.007	4.53E-09	349376	0.0000996	35
rs200062544	7:17260246	0.47	A	0.049	0.007	2.64E-12	349376	0.00014	49
rs2315024	19:19423817	0.33	A	0.0434	0.0073	2.98E-09	349376	0.000101	35
rs2465018	6:51241140	0.23	A	0.0635	0.0082	1.38E-14	349376	0.000172	60
rs2472297	15:75027880	0.27	T	0.1576	0.0078	3.82E-91	349376	0.00117	408
rs28548701	15:74346021	0.8	C	–0.0502	0.0086	5.82E-09	349376	0.0000975	34
rs28676340	15:75449794	0.16	G	–0.0564	0.01	1.96E-08	349376	0.000091	32
rs34591452	15:74492585	0.24	T	0.0759	0.0081	5.48E-21	349376	0.000251	88
rs34606716	7:75820449	0.24	A	–0.0453	0.0082	2.7E-08	349376	0.0000873	31
rs3815455	7:75611756	0.29	T	0.0647	0.0076	1.74E-17	349376	0.000207	72
rs397074	15:74599997	0.31	C	–0.0521	0.0075	2.8E-12	349376	0.000138	48
rs4410790	7:17284577	0.63	C	0.1215	0.0072	1.89E-64	349376	0.000814	285
rs4817505	21:34343828	0.39	C	0.0411	0.0071	6.22E-09	349376	0.0000959	34
rs4887165	15:74889356	0.81	C	0.0539	0.0089	1.22E-09	349376	0.000105	37
rs60223362	7:17459648	0.2	C	–0.0747	0.0086	5.35E-18	349376	0.000216	75
rs6495129	15:75196717	0.2	T	–0.0582	0.0086	1.35E-11	349376	0.000131	46
rs6697410	1:26756209	0.74	T	0.0436	0.0079	4.1E-08	349376	0.0000872	30
rs6965666	7:17177312	0.28	C	–0.0503	0.0078	9.14E-11	349376	0.000119	42
rs7174381	15:75613289	0.31	C	0.0522	0.0075	3.85E-12	349376	0.000139	48
rs73071153	7:17545964	0.03	A	–0.1312	0.0194	1.32E-11	349376	0.000131	46
rs73075157	7:17566844	0.13	A	–0.0678	0.0103	5.42E-11	349376	0.000124	43
rs73169830	22:24885208	0.08	C	0.1027	0.0131	3.81E-15	349376	0.000176	61
rs73424602	22:41461176	0.4	T	–0.0432	0.007	7.84E-10	349376	0.000109	38
rs77821156	7:17331450	0.11	G	0.0643	0.0113	1.39E-08	349376	0.0000927	32
rs79217743	15:75117912	0.14	T	–0.0602	0.0102	3.34E-09	349376	0.0000997	35
rs79413667	7:17399486	0.03	G	–0.1171	0.0201	6.03E-09	349376	0.0000971	34
rs79694830	7:17286087	0.06	T	0.0951	0.015	2.26E-10	349376	0.000115	40
rs7999399	13:89233505	0.55	T	0.0379	0.0069	4.96E-08	349376	0.0000863	30
rs9624470	22:24820268	0.58	A	0.0729	0.007	3.06E-25	349376	0.00031	108

SNP, single-nucleotide polymorphism; EAF, effect allele frequency; EA, effect allele; SE, standard error. R^2^ was calculated as follows: 2*beta^2^*EAF*(1-EAF)/(2*beta2*EAF*(1-EAF) + se^2^*2*N*EAF(1-EAF)). The F-statistic for each SNP was calculated as follows: F = (N − 2)*R^2^/(1 − R^2^).

### Genetic Summary Data of Kidney Function

We extracted summary statistics for CKD and eGFR from the CKDGen consortium. The meta-analyses of GWAS of the kidney function comprised 43 studies of European ancestry (*n* = 117,165, 12,385 cases) (22). Participants were diagnosed with CKD GFR categories 3–5 (G3–G5) based on eGFR < 60 ml/min/1.73 m^2^ (23). Except the two studies that reported data, all definitions of CKD G3-G5 came from a single assessment of eGFR (22). eGFR is calculated by the Schwartz formula (<18 years) (24) and the Chronic Kidney Disease Epidemiology Collaboration equation for adults (>18 years) (25). Wuttke et al. reported the characteristics of the CKDGen alliance study (26). Albuminuria was also extracted from the CKDGen consortium, which enrolled a total of 54,116 participants of European ancestry (27). Albuminuria is defined as a condition where the urinary albumin–creatinine ratio is >17 mg/g (>1.92 mg/mmol) in men and >25 mg/g (>2.83 mg/mmol) in women (27). Sex differences in albuminuria were obtained from the study of Warram et al. (28). All summary data can be obtained from the UK Medical Research Council Integrative Epidemiology Unit Open GWAS Project database.^3^

All research analyses were based on publicly available GWAS summary statistics, and no additional ethics approval and informed consent were required.

### Statistical Analyses

The conventional MR method was applied in this study^4^ (29). The fixed-effect inverse-variance-weighted (IVW) model was used to examine a causal association, and it was considered as the main analytical method (30, 31). The IVW method included individual MR effects of SNPs to derive overall weighted effects of the potential causal association. Multiplicative random IVW was used as a supplement if necessary. Furthermore, the forest plots showed the MR-derived odds ratio (OR) of CKD and albuminuria, and log odds of eGFR were predicted by genes when an additional cup of tea is consumed daily. “Leave-one-out” analyses were performed to determine whether a causal association was reliant on any single SNP. Causal Analysis Using Summary Effect estimates (CAUSE) account for uncorrelated and correlated pleiotropy simultaneously (32).

The IVW method assumes that all genetic variants satisfy the three assumptions of the instrumental variables: (1) closely associated with tea intake; (2) not associated with confounders of the association between tea intake, CKD, eGFR, and albuminuria; and (3) risk factors associated with the risks of CKD, eGFR, and albuminuria, which could only be induced *via* tea consumption (33, 34). *F*-statistics were used to test weak instrumental variables, *F*-statistics = (*N* − 2) × *R*^2^/(1 − *R*^2^), where *R*^2^ is the variance in tea consumption explained by the genetic instrument, *k* is the number of genetic variants, and *n* is the sample size. *F* > 10 proved to be a strong genetic instrument in the MR study. Cochran’s *Q* test was used to quantify the size of heterogeneity effect between the genetic instruments (35), which might indicate that the potential horizontal pleiotropy violated the third MR assumption. Potential violation of the second and third MR assumptions was tested using several approaches, such as the MR-Egger regression (36) and the weighted median (37) methods. The intercept from MR-Egger also provides a formal test for directional pleiotropy. The association between the selected SNPs and exposures was validated in the PhenoScanner database^5^ (Supplementary Table 1). SNPs associated with the traits other than tea intake were recorded at the significance level (*p* < 5 × 10^–8^).

MR-Egger is an adaption of Egger regression, which realizes directional pleiotropy by introducing an intercept in the weighted regression model. When the value of the intercept term is away from 0, it indicates horizontal pleiotropy (38). Based on this approach, unbiased estimates are performed in the presence of pleiotropic instruments, assuming that the magnitude of pleiotropic effects is independent of the size of the instrumental variables—SNPs associated with tea intake (38).

The weighted median method uses each instrumental variable to weigh the estimated value of the reciprocal of its variance to rank them in MR. The median result is selected, and the single MR estimated value and CIs based on the bootstrapping technique are displayed (39). The weighted median requires and assumes that at least half of the instruments are effective (40).

Steiger-MR was used to test whether the SNPs explained significantly more variance in exposure than the outcome (the opposite may indicate reverse causation) (41).

Bonferroni correction (*p* = 0.05/3 outcomes/3 methods) was applied to adjust multiple testing (*p* = 0.006) in univariable MR. The “TwoSampleMR” package (version 0.5.5), “CAUSE” package (version 1.2.0), and R software version 3.6.1 were used for all statistical analyses.

## Results

### Causal Association Between Tea Consumption and CKD G3-G5

**FIGURE 1:**
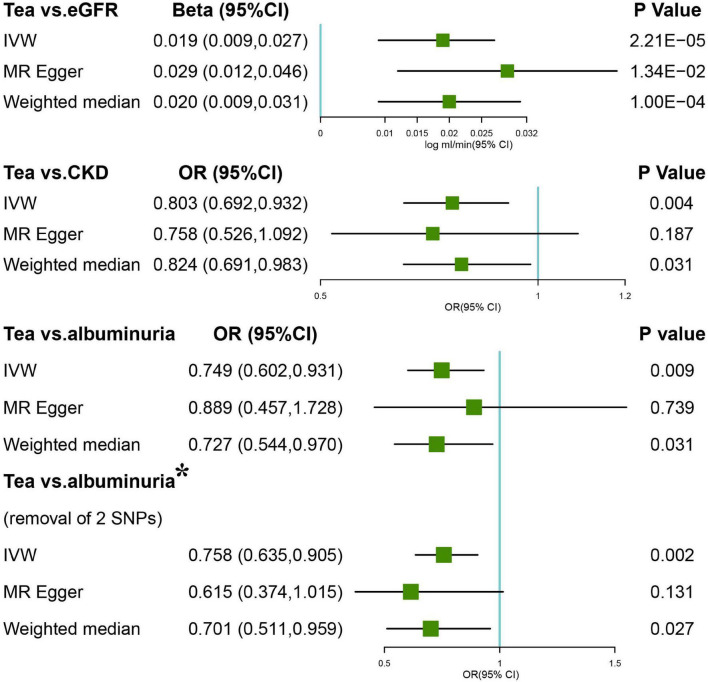
Forest plots of Mendelian randomization (MR) study using genetically predicted tea consumption with chronic kidney disease (CKD), glomerular filtration rate (GFR), and albuminuria. Inverse-variance-weighted (IVW), MR-Egger, weighted median, simple, and weighted mode were used in this study. *Denotes the removal of two single-nucleotide polymorphism (SNPs; rs1030510 and rs4410790) that might give rise to significant heterogeneity (MR-Egger intercept *p* = 0.02).

**FIGURE 2:**
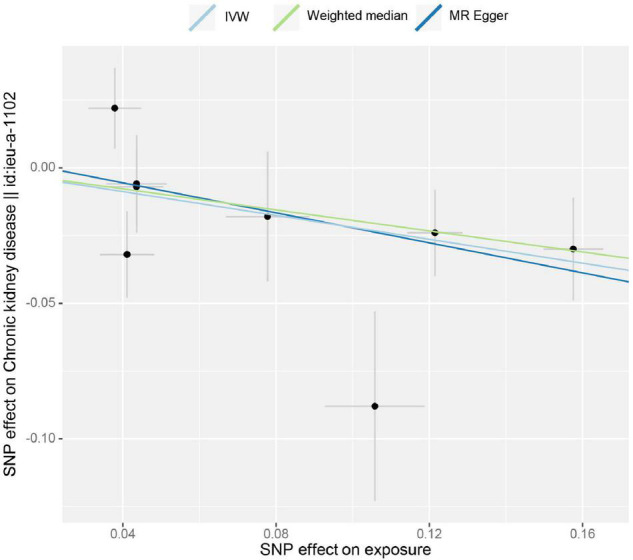
Scatter plot of the effect size for each SNP on tea consumption and CKD G3–G5.

The estimation of the causal effect of tea consumption on CKD G3-G5 from the MR analyses is shown in Figure 1. In the IVW MR analyses, the OR of CKD for an additional daily cup of tea was 0.803 (*p* = 0.004). The MR-Egger test detected no directional pleiotropy (*p* = 0.732). In the leave-one-out analyses, the estimates ranged from 0.779 to 0.830, suggesting that the observed result was not the effect of a single SNP (Supplementary Figure 1). The weighted median analysis (OR = 0.824; *p* = 0.031) of the estimated values was concordant and similar in size, but it could not confirm the protective effect of tea against CKD G3-G5 in the adjusted value of *p* (Figure 1). The estimates from the MR-Egger (OR = 0.758; *p* = 0.187) were not statistically significant, suggesting limited effectiveness. Additionally, there was no indication of pleiotropy when the intercept was derived from the MR-Egger regression (Egger intercept: 0.006, the value of *p*: 0.732). The value of *p* for the MR-Egger method was 0.146, which also suggested no significant sign of heterogeneity. The causal effect of tea intake on the CKD estimated by CAUSE was unsupported (*p* = 0.132). The scatter plot of the SNP—CKD associations against SNP—tea associations is shown in Figure 2. The forest plots of tea—CKD estimates in each SNP are presented in Supplementary Figure 2.

### Causal Association Between Tea Consumption and eGFR

**FIGURE 3:**
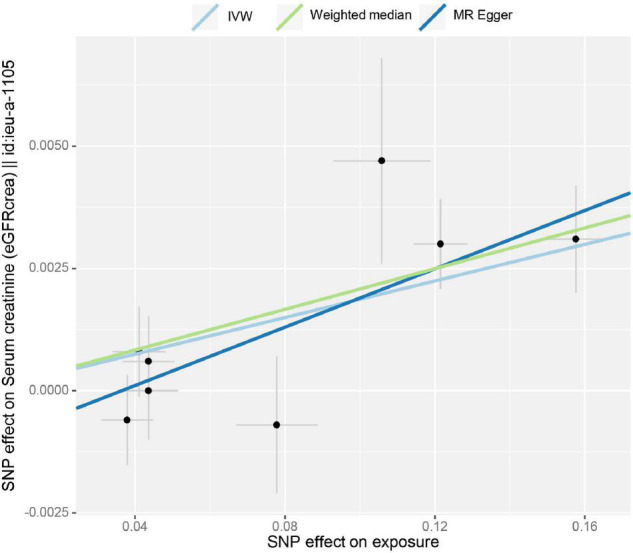
Scatter plot of the effect size for each SNP on tea consumption and the estimated glomerular filtration rate (eGFR).

Inverse-variance-weighted analyses of tea consumption and eGFR provided correlative evidence for an association (β = 0.019 log ml/min/1.73 m^2^ per cup per day; *p* = 2.21 × 10^–5^) (Figure 1). In the leave-one-out analyses, β ranged from 0.015 to 0.020 (Supplementary Figure 3). There was no evidence of directional pleiotropy (MR-Egger intercept *p* = 0.183) and horizontal pleiotropy (heterogeneity *p* = 0.521). This was consistent with the estimates from the weighted median (β = 0.020; *p* = 1.00 × 10^–4^) and MR-Egger (β = 0.029; *p* = 1.34 × 10^–2^) analyses (original *p* < 0.05; adjusted *p* > threshold value), which were more robust to pleiotropy (Figure 1). The causal effect of tea intake on eGFR estimated by CAUSE was insignificant (*p* = 0.141). The scatter plot of the SNP—eGFR associations against the SNP—tea associations is shown in Figure 3. The forest plot of tea—eGFR estimates in each SNP is presented in Supplementary Figure 4.

### Causal Association Between Tea Consumption and Albuminuria

**FIGURE 4:**
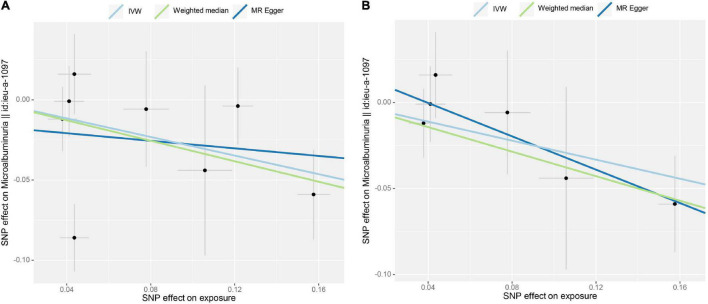
Scatter plot of the effect size for each SNP on tea consumption and albuminuria. **(A)** No SNP was excluded. **(B)** Two SNPs were removed (rs1030510 and rs4410790) due to potential horizontal pleiotropy.

Initial IVW analyses of tea consumption and albuminuria did not provide strong evidence for their correlation (OR = 0.749; *p* = 0.009) (Figure 1). There was evidence of directional pleiotropy (MR-Egger intercept *p* = 0.019) and no horizontal pleiotropy (heterogeneity *p* = 0.575). Based on the leave-one-out method, these outlying SNPs (rs1030510 and rs4410790), which mainly caused heterogeneity, were removed. Then, through the random-effect IVW (OR = 0.758; *p* = 0.002) and weighted median (OR = 0.701; *p* = 0.027) methods (original *p* < 0.05; adjusted *p* > threshold value), the estimate of the causal effect of tea consumption on albuminuria was similar in direction and magnitude to CKD G3-G5 (Figure 1). In the leave-one-out analyses, ORs ranged from 0.723 to 0.894, showing a consistency in the entire estimate (Supplementary Figure 5). There was neither significant horizontal pleiotropy (heterogeneity *p* = 0.893) nor directional pleiotropy (MR-Egger test *p* = 0.383). The scatter plot of the SNP—albuminuria associations against the SNP—tea associations before and after removing two SNPs is shown in Figures 4A,B. The causal effect of tea intake on albuminuria estimated by CAUSE was insignificant (*p* = 0.620). The forest plots of tea—eGFR estimates in each SNP after removing outlying SNPs are presented in Supplementary Figure 6.

### Sensitivity Analyses

A GWAS involving CKD diagnosis in European men and women from the NHGRI-EBI GWAS Catalog^6^ was used to further validate the reliability of results. The MR analysis was conducted using SNP sites associated with CKD exposure in EBI, indicating the similar causal associations of the diagnosed CKD with IVW (OR = 0.803, *p* = 0.004). However, weighted median (OR = 0.824, *p* = 0.039) and MR Egger (OR = 0.758, *p* = 0.187) could not confirm a casual association (Supplementary Figure 7).

The Steiger-MR analysis was used to identify the presence of horizontal pleiotropy and the robustness of the causal effect estimates. There was no sign of heterogeneity, and Steiger-MR indicated that the SNPs explained more variance in exposure than the outcome (*p* > 0.05) (e.g., CKD, eGFR, and albuminuria).

## Discussion

This two-sample MR study used summary-level data from UK Biobank and the CKDGen consortium to estimate the potential causal association of tea consumption with CKD, eGFR, and albuminuria. A total of 2,672 SNPs associated with tea consumption were found in UK Biobank, and 45 independent and available SNPs were found in CKDGen. The MR analyses showed that an increase in tea consumption appeared to be protective against CKD, eGFR, and albuminuria. The effects were generally similar in magnitude across diverse sensitivity analyses. Therefore, tea intake may be beneficial to renal function.

The association between tea intake and the risk of CKD remains inconsistent in previous longitudinal and cross-sectional studies. In an Iranian longitudinal study covering 1,780 adult Iranians (2006–2008 to 2012–2014), high tea intake was not associated with the risk of CKD (12). In the Doetinchem Cohort Study, including 4,722 individuals aged 26–65 years, tea consumption was not significantly associated with the changes in eGFR (14). However, in another Spanish cohort, including 5,851 overweight/obese elderly with MetS, overweight/obese adults with Mets, and high tea intake (>at least 1 cup/day) had a greater decline in eGFR in 1 year (13). Apart from this, the relationship between the intake of different types of tea (black tea, oolong tea, or green tea) and kidney function is also heterogenous. A Guangzhou Biobank cohort study including 12,428 elderly people did not find a significant association of green or black tea with eGFR. Nevertheless, a negative association between an intake of oolong tea and eGFR was found (15). The Singapore Chinese Health Study, a prospective cohort study of 63,257 participants aged 45–74 years, identified that tea intake is not associated with the risk of ESRD over an average follow-up of 17 years (16). Compared with previous studies, our study found that genetically predicted tea intake was causally associated with the decrease of CKD and albuminuria and the increase of eGFR. This MR study, in which confounding bias and reverse causality were avoided and a large number of individuals were from two-sample designs, is different from previous observational studies.

Several potential biological mechanisms may underlie an inverse association between tea intake and the risk of renal impairment. Growing evidence has shown that the generation of reactive oxygen species (ROS) plays an important role in the pathogenesis of kidney disease (42). Several interventional studies in humans have reported that the consumption of black and green tea improves vasodilator effects and decrease ROS concentrations in patients with renal failure (43, 44). Ardalan et al. reported that short-term intake of black tea could improve endothelial function and endothelium-dependent arterial vasodilation in renal transplant recipients (43). Hsu et al. revealed that the decaffeinated green tea extract (catechins) reduced hemodialysis-induced production of hydrogen peroxide and hypochlorous acid, the risk factors of atherosclerotic disease, and proinflammatory substances (44).

Previous studies have shown that tea catechins improve the metabolic mechanism of endothelial function. For example, the most abundant catechins in tea are epigallocatechin-3-gallate (EGCG), epigallocatechin, and epicatechin (45). These substances have been identified to be fairly strong antioxidants and free radical scavengers (46). Additionally, some murine and *in vitro* studies found the potential effects of EGCG on renal function. Yamabe et al. suggested that EGCG may reduce urinary protein excretion and serum glucose in mice with diabetic nephropathy (47). In rodent models with induced renal failure, tea catechins (including EGCG) have shown uniquely beneficial effects in decreasing nephrotoxicity (48, 49). The potential signal pathway mechanisms demonstrated that tea catechins could increase antioxidant activity, prostaglandin levels in the kidney, changes in transforming growth factor-β1 expression, and the regulation of nuclear factor-κβ (47, 48, 50, 51). Furthermore, compared with black tea, green tea has shown positive associations with renal function. The intake of green tea has been reported to reduce urinary oxalate excretion and deposition in rat models (52). In addition, green tea is very rich in catechins, including EGCG, which exerts an *in vivo* antiproliferative effect on renal cell carcinoma cell lines (53). Chinese green tea (CGT) has also been demonstrated for its protection against ROS, which causes apoptosis, inflammation, and damage in the lung tissue, among rats exposed to tobacco smoke for long periods; at the same time, CGT could also relieve nicotine toxicity, exerting antioxidant and anti-inflammatory properties (54, 55). Caffeine intake has been identified to exacerbate renal failure in a rat model (56). A cup of pure green tea usually contains around 25 mg of caffeine per 8 oz serving, which is considered to be a low amount (57). It is roughly one-fourth of the amount of caffeine in a typical cup of coffee and roughly half of the amount of caffeine in a typical cup of black tea (58).

The main advantage of this study is the two-sample method, which has a large summary-level genetic data that is able to avoid potential confounding factors and reverse causation in observational studies. The second advantage is that genetically predicted consumption of tea has been identified in a large GWAS of 349,376 European individuals, to diminish weak instrument bias (*F*-statistic > 10). However, this study has some limitations. First, the results did not apply to other populations on account of deviations from the data limited in European populations. Second, there were no non-linear relationships or stratification effects due to the summary-level data. Lastly, the type of tea and the amount of intake are important for exploring their causal association with the exposed genes. Further studies should take into consideration the overall impact of tea consumption.

## Conclusion

It can be genetically predicted that there is a causal relationship of an extra cup of tea a day with the reduced risk of CKD and albuminuria and the increased level of eGFR. This MR study conducted a complete and detailed analysis of tea intake and renal function, which provided new evidence for the prevention and treatment of CKD.

## Data Availability Statement

The datasets presented in this study can be found in online repositories. The names of the repository/repositories and accession number(s) can be found in the article/Supplementary Material.

## Ethics Statement

Ethical review and approval were waived for this study, all the data from Mendelian randomization is publicly accessible. Informed consent was obtained from all subjects in the original genome-wide association studies. The patients/participants provided their written informed consent to participate in this study.

## Author Contributions

YZ, YX, WW, TW, SS, JY, XW, LC, HZ, QY, and XL designed the study. YX analyzed the data. YZ wrote the manuscript. All authors contributed to the article and approved the submitted version.

## Conflict of Interest

The authors declare that the research was conducted in the absence of any commercial or financial relationships that could be construed as a potential conflict of interest.

## Publisher’s Note

All claims expressed in this article are solely those of the authors and do not necessarily represent those of their affiliated organizations, or those of the publisher, the editors and the reviewers. Any product that may be evaluated in this article, or claim that may be made by its manufacturer, is not guaranteed or endorsed by the publisher.

## Footnotes

1
http://www.nealelab.is/uk-biobank

2
https://github.com/Nealelab/UK_Biobank_GWAS

3
http://gwas.mrcieu.au.uk

4
https://mrcieu.github.io/TwoSampleMR/articles/perform_mr.html#mr-methods-1

5
http://www.phenoscanner.medschl.cam.ac.uk/

6
https://www.ebi.ac.uk/gwas/downloads/summary-statistics
