# Supplementary material for: Causal Association Between Tea Consumption and Kidney Function: A Mendelian Randomization Study
Source: Front Nutr. 2022 Mar 29;9:801591. doi: 10.3389/fnut.2022.801591 (PMC9002236; doi:10.3389/fnut.2022.801591)
Supplement: Supplementary file 1 [file Data_Sheet_5.docx]

**Table 1S.** Potential confounders associated with selected SNPs.

| rs199621380 | Neutrophil percentage of granulocyte；Neutrophil percentage of white cells；Granulocyte percentage of myeloid white cells |
| --- | --- |
| rs11022751 | Body mass index；Diastolic blood pressure；Age at menarche |
| rs10741694 | Hypertension |
| rs397074 | Hypertension |
| rs2472297 | Creatinine in urine；Platelet distribution width |
| rs79217743 | Granulocyte percentage of myeloid white cells；Diastolic blood pressure |
| rs7174381 | Creatinine in urine |
| rs12600469 | Weight |
| rs2315024 | Self-reported high cholesterol |
| rs9624470 | Creatinine in urine |
| rs73424602 | Serum urate；Uric acid |
| rs1481012 | Cholesterol ldl |
| rs4410790 | Creatinine in urine |
| rs3815455 | Creatinine in urine |


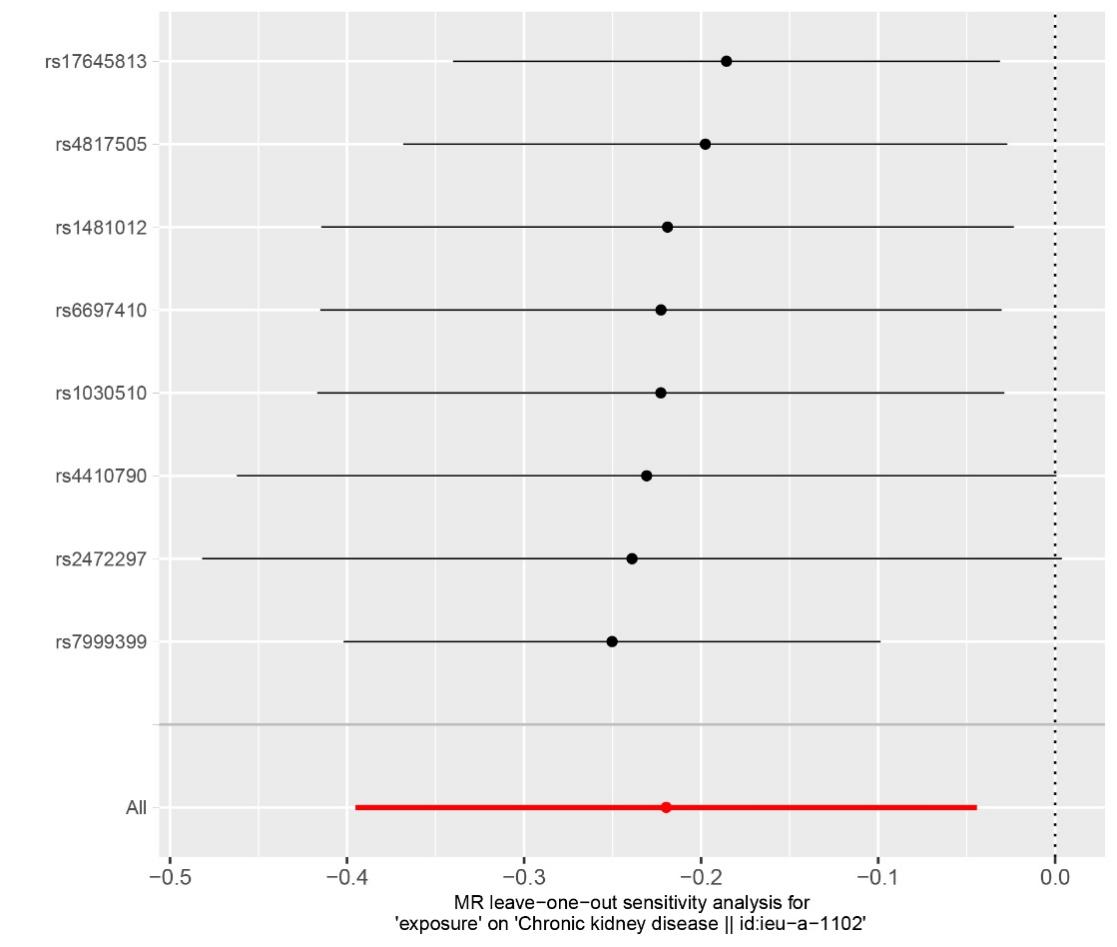


**Figure 1S.** Leave-one-out sensitivity analysis for the CKD G3-G5 using SNP-associated tea consumption.


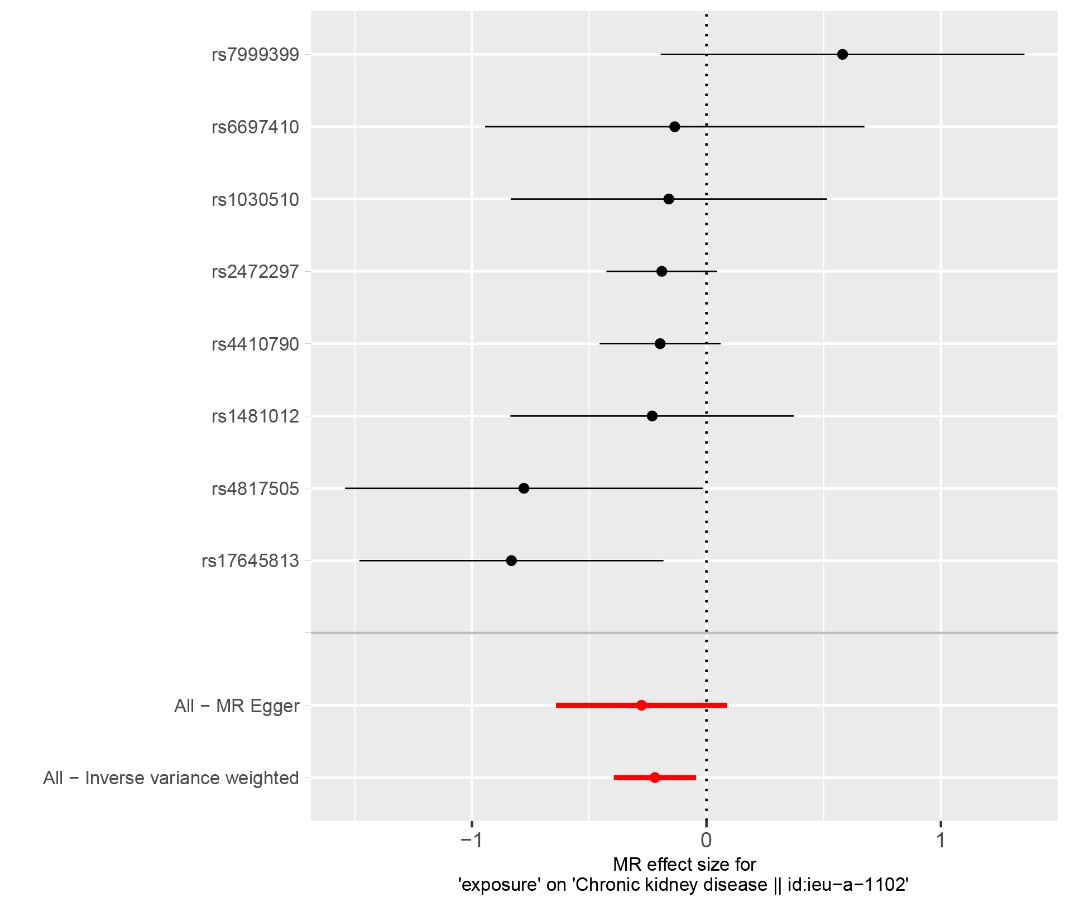


**Figure 2S.** The forest plot for MR analyses of causal associations between each tea

SNP and CKD


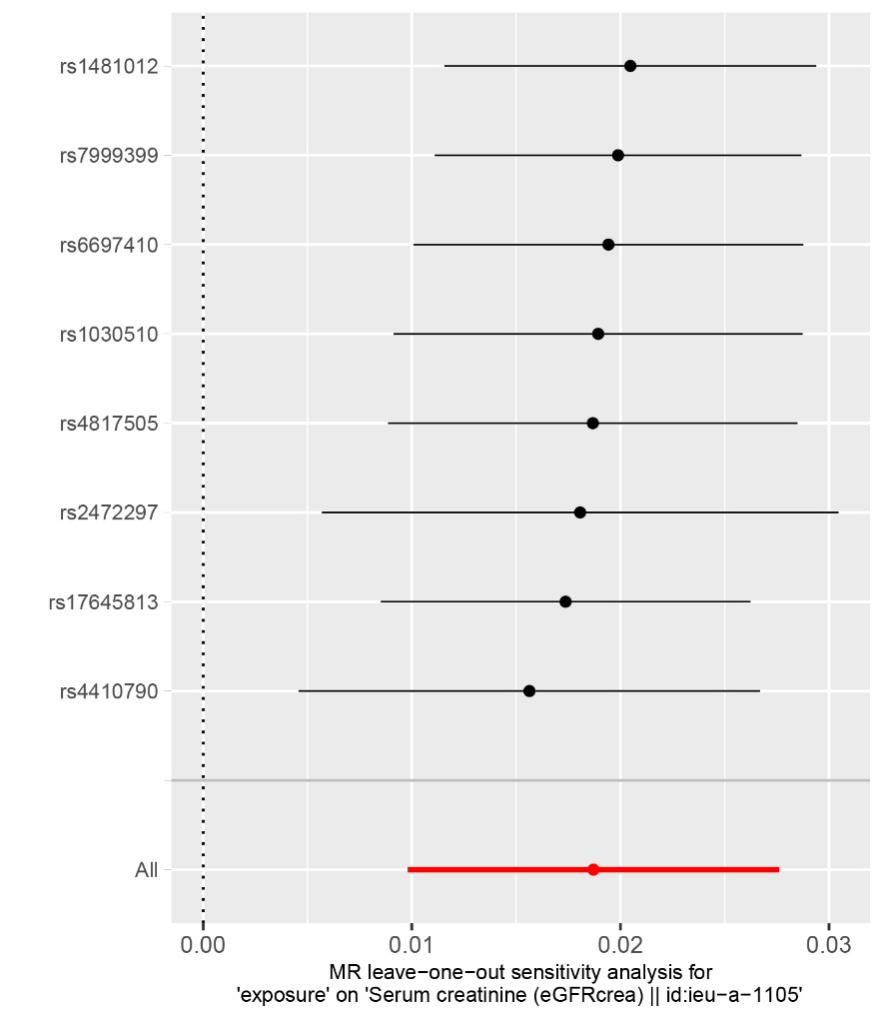


**Figure 3S.** Leave-one-out sensitivity analysis for the eGFR using SNP-associated tea consumption.


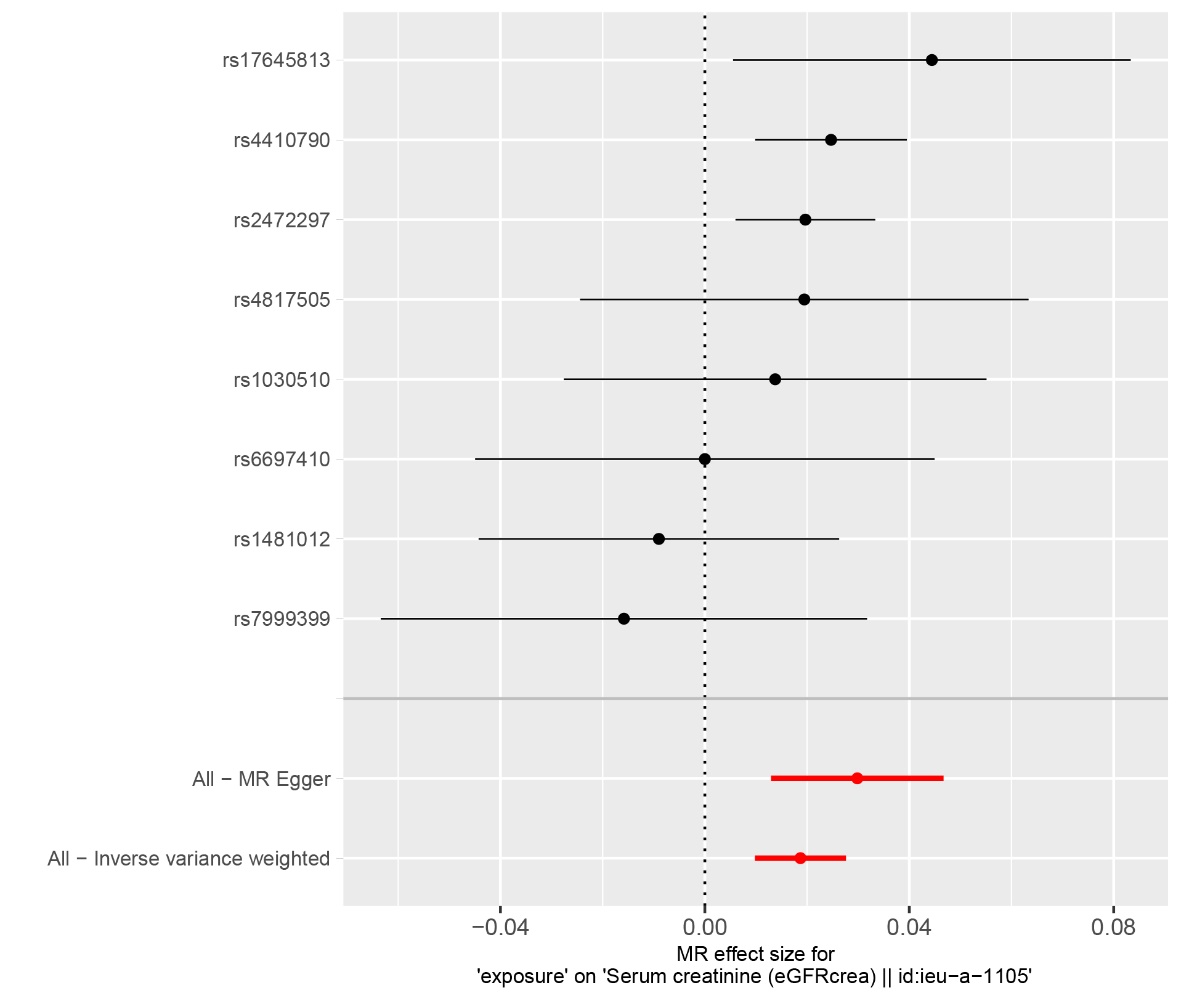


**Figure 4S.** The forest plot for MR analyses of causal associations between each tea

SNP and eGFR.


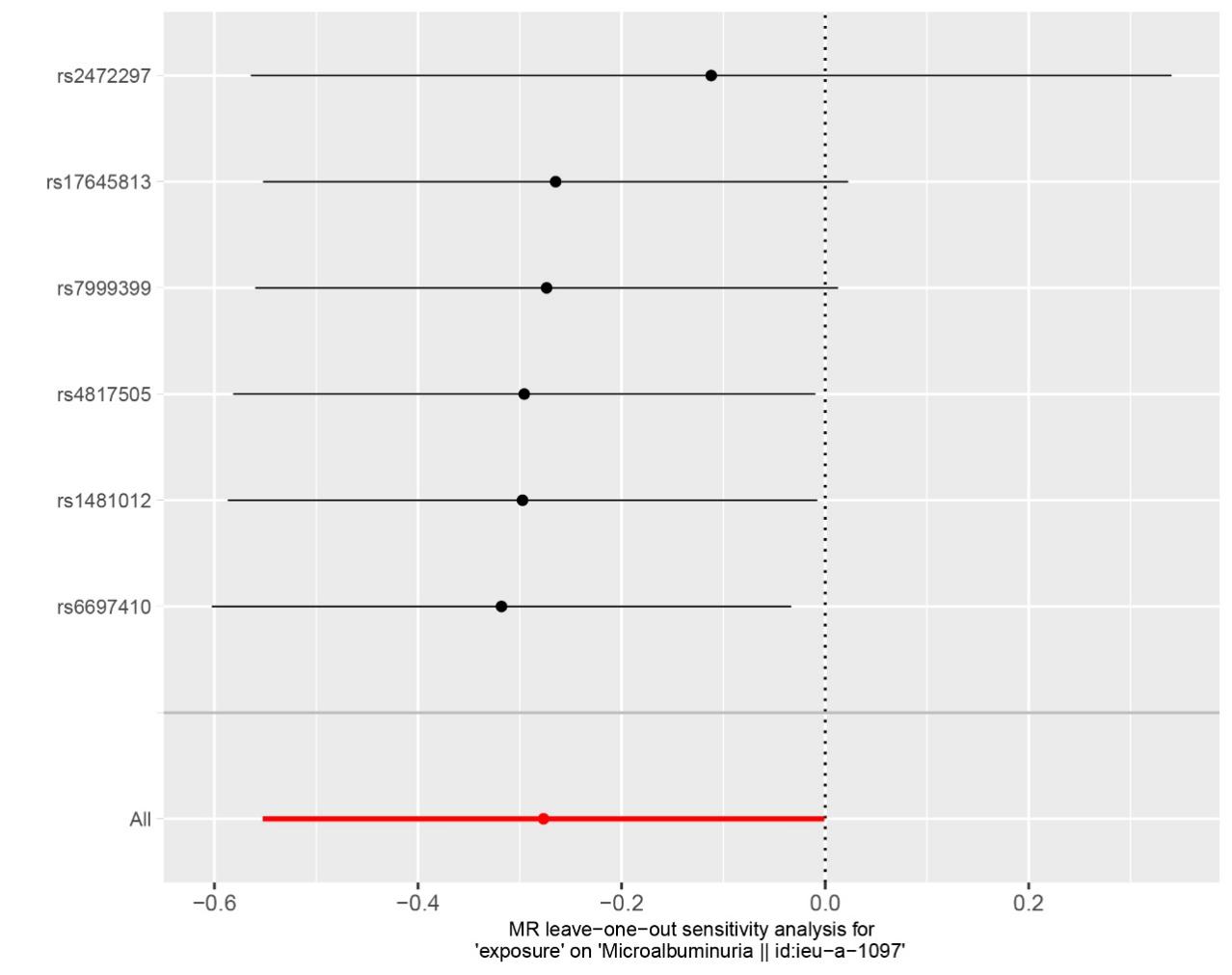


**Figure 5S.** Leave-one-out sensitivity analysis for the albuminuria using SNP-associated tea consumption after removal of 2 SNPs.


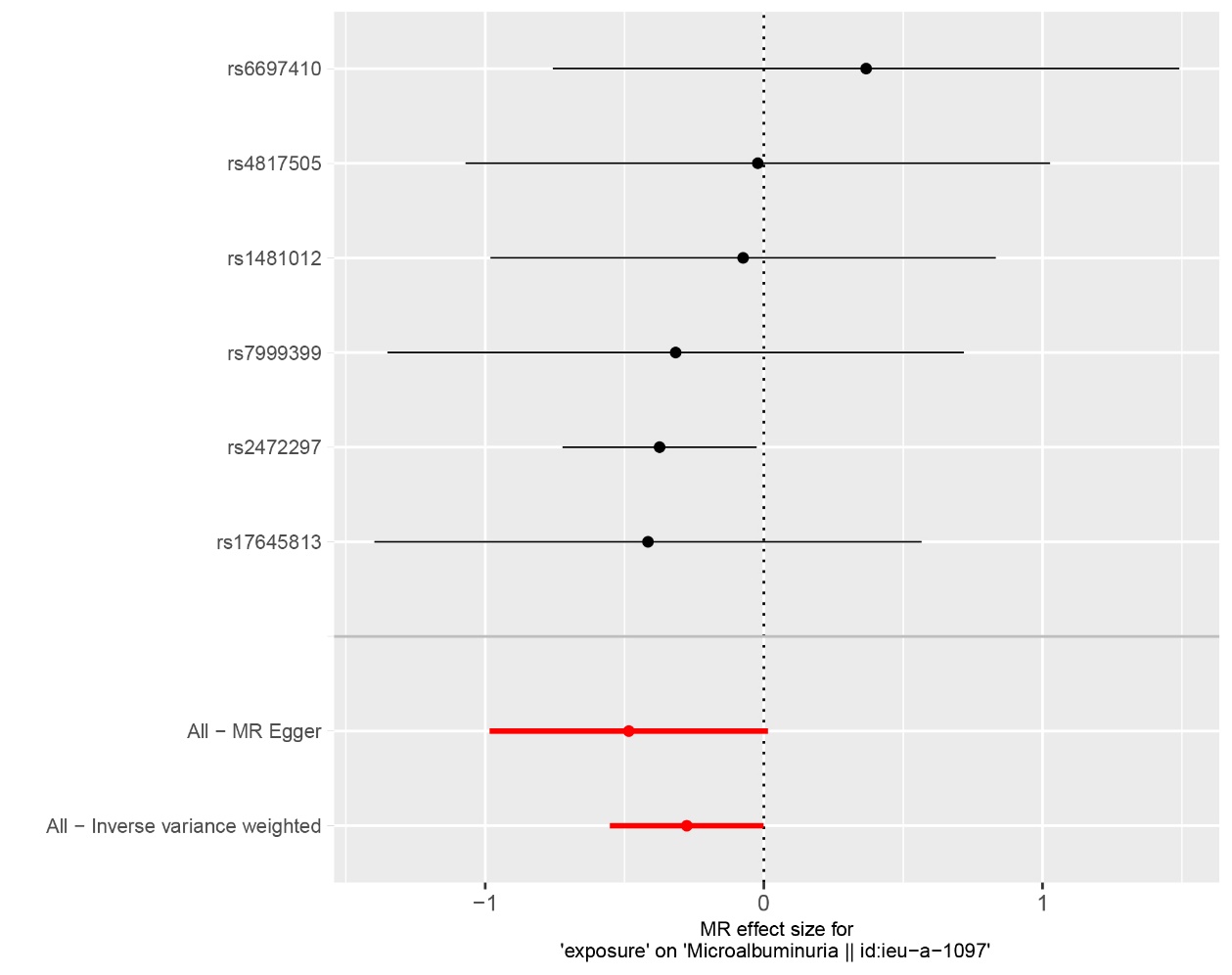


**Figure 6S**. The forest plot for MR analyses of causal associations between each tea

SNP and albuminuria after removal of 2 SNPs.


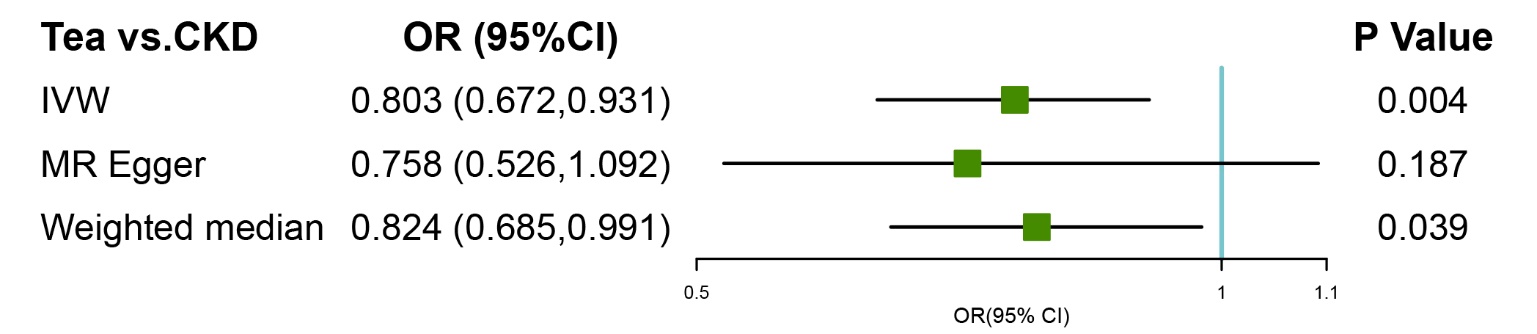


**Figure 7S.** Forest plots of MR study using genetically predicted tea consumption with chronic kidney disease (CKD) from the NHGRI-EBI Catalog of human genome-wide association studies
